# Slope position- mediated soil environmental filtering drives plant community assembly processes in hilly shrublands of Guilin, China

**DOI:** 10.3389/fpls.2022.1074191

**Published:** 2023-01-06

**Authors:** Kunquan Chen, Yuanfang Pan, Yeqi Li, Jiaying Cheng, Haili Lin, Wenhua Zhuo, Yan He, Yaocheng Fang, Yong Jiang

**Affiliations:** ^1^ Key Laboratory of Ecology of Rare and Endangered Species and Environmental Protection (Guangxi Normal University), Ministry of Education, Guili, China; ^2^ Guangxi Mangrove Research Center, Guangxi Academy of Sciences, Beihai, Guangxi, China

**Keywords:** community assembly, plant functional traits, soil poperties, slope positions, environmental filtering

## Abstract

**Background and aims:**

A major goal of community ecology focuses on trying to understand how environmental filter on plant functional traits drive plant community assembly. However, slopes positions- mediated soil environmental factors on community-weighted mean (CWM) plant traits in shrub community has not been extensively explored to analyze and distinguish assembly processes.

**Methods:**

Here, we surveyed woody shrub plant communities from three slope positions (foot, middle, and upper) in a low hilly area of Guilin, China to assess differences in functional trait CWMs and environmental factors across these positions. We also measured the CWMs of four plant functional traits including specific leaf area, leaf dry matter content, leaf chlorophyll content, and leaf thickness and nine abiotic environmental factors, including soil water content, soil organic content, soil pH, soil total nitrogen, soil total phosphorus, soil total potassium, soil available nitrogen, soil available phosphorus, and soil available potassium. We used ANOVA and Tukey HSD multiple comparisons to assess differences in functional trait CWMs and environmental factors across the three slope positions. We used redundancy analysis (RDA) to compare the relationships between CWMs trait and environmental factors along three slope positions, and also quantified slope position-mediated soil environmental filtering on these traits with a three-step trait-based null model approach.

**Results:**

The CWMs of three leaf functional traits and all soil environmental factors except soil pH showed significant differences across the three slope positions. Soil total nitrogen, available nitrogen, available potassium, and soil organic matter were positively correlated with the CWM specific leaf area and leaf chlorophyll content along the first RDA axis and soil total potassium, total phosphorous, and soil water content were positively correlated with the CWM leaf dry matter content along the second RDA axis. Environmental filtering was detected for the CWM specific leaf area, leaf dry matter content, and leaf chlorophyll content but not leaf thickness at all three slope positions.

**Conclusions:**

Ultimately, we found that soil environmental factors vary along slope positions and can cause variability in plant functional traits in shrub communities. Deciduous shrub species with high specific leaf area, low leaf dry matter content, and moderate leaf chlorophyll content dominated at the middle slope position, whereas evergreen species with low specific leaf area and high leaf dry matter content dominated in slope positions with infertile soils, steeper slopes, and more extreme soil water contents. Altogether, our null model approach allowed us to detect patterns of environmental filtering, which differed between traits and can be applied in the future to understand community assembly changes in Chinese hilly forest ecosystems.

## Introduction

Plant functional traits are defined as the morpho-physio-phenological plant features that indirectly affect fitness within a given environmental context that also provide insights into how environmental factors shape species distributions at regional and local scales (Violle et al., 2007; de Bello et al., 2013). Functional traits can improve biodiversity predictions with environmental change because they represent a species’ strategy use under different resources (de Bello et al., 2013; Bruelheide et al., 2018; Maes et al., 2020). For example, specific leaf area and leaf dry matter content are core traits in leaf economic spectrum and nutrient resources (Hodgson et al., 2011; Gao et al., 2022; Ren et al., 2022). Specially, nutrient-poor soils are often associated with leaf economic spectrum of “slow-growing” plants with older, smaller specific leaf area with high leaf dry matter content (Ordoñez et al., 2009). In contrast, “fast-growing” plants that are often associated with nutrient-rich soils possess the opposite sets of traits (i.e. younger leaves, larger specific leaf area, higher leaf nitrogen; Wright et al., 2001; Wright et al., 2004). With these traits, specific leaf area and leaf dry matter content are mainly affected by soil nutrient factors, such as pH and soil nitrogen and phosphorus content (Wang et al., 2022). Generally, soil total nitrogen content and the ratio of carbon to nitrogen are positively correlated with specific leaf area and negatively correlated with leaf dry matter content (Hodgson et al., 2011). Additionally, leaf chlorophyll content provides information about the physiological state of the plant (Lu et al., 2018), where any shortage of soil water or nitrogen significantly reduces its chlorophyll and photosynthetic rate (Qiu et al., 2018). Leaf thickness also demonstrateds the physical resistance of the leaf blade to adverse environmental condititons (Afzal et al., 2017; Pinho et al., 2018), and has been shown to be positively correlated with soil fertility and soil water availability (Schmitt et al., 2022).

Understanding the deterministic processes that underly the functional structure of ecological communities is one of the central goals of community ecology (Cornwell and Ackerly, 2009; de Bello et al., 2013). In theory, the environmental filtering hypothesis proposed that abiotic environment factors act as “filters” to select species from the regional pool with similar trait values into communities to adapt to the local environment conditions (Kraft et al., 2015; Le Bagousse-Pinguet et al., 2017). A substantial number of studies have shown that community composition is often influenced by environment-driven factors that results in trait convergence—particularly at local scales (Alice et al., 2018; Báez et al., 2022; Da et al., 2022). Habitats in the slope foot tend to be more moist than at higher, steeper positions, which are often more dry and exposed, where furthermore, these temperature and moisture differences may alter nutrient mineralization along the slope position. Altogether, these environmental differences can cause flexible responses in plant functional traits, with the most researched being tree height, specific leaf area, and leaf dry matter content (Niinemets et al., 2006; Mitchell et al., 2016; Guo et al., 2018). For instance, Lim et al. (2017) demonstrated that the community-weighted mean (CWM) height decreased down a slope into frost hollows while the CWM the leaf area was greater toward the top of the slope position. Additionally, Méndez-Toribio et al. (2017) found that wood density and leaf dry matter content were greater at lower slope positions. Although several previous studies have shown links between topography and soil resource availability, soil environmental factors along different slope positions have not been extensively investigated to understand how differences in plant functional traits affect shrub communities (Punchi-Manage et al., 2012; Liu et al. (2014).

Shrubs act as an essential component of global and regional vegetation and have rich characteristic such as wide distributions, high germination rates, and high morphological and physiological plasticity, which is an ideal natural experimental model to facilitate adaptation for diverse environments (Xu et al., 2020; Venn et al., 2021). Generally, shrubs are an important component of forest ecosystems and play a vital role in natural forest dynamics, especially during the first stages of tree development. Moreover, shrubs have gained importance since they are more tolerant to drought and aridity than tree species, which has become significant under the current context of global warming (Bateman et al., 2018). Despite the importance of shrubs on the global scale, they are often understudied studied compared to other plants, like trees. Because of these issues, it is necessary to identify the response of shrubs to environmental conditions based on the perspective of functional traits at the local community level. Currently, there are many studies that have focused on trying to understand changes in communities level plant features over natural gradients to assess community assembly processes, such as trait convergence, trait divergence, and stochastic process. Pinho et al., 2018 found soil fertility operates as a key filter causing functional convergence towards more conservative resource-use strategies in tropical forest regeneration. Da et al. (2022) demonstrated that soil and topography factors were the main environment driver of trait convergence in temperate forest communities at local scale. However, very few have focused on trait convergence by habitat filtering for shrub plants in different environments, or how these traits could be affected by harsh conditions due to complex abiotic filters (e.g., soil and slope positions factors; Asefa et al., 2017; Molina-Venegas et al., 2018).

In this study, we measured functional traits of shrubs along a slope gradient in the Jiangjiaba hilly area of Guilin, China to assess relationships between community-level trait values and environmental factors. Initially, we expected slope position to interact with environmental factors to filter trait values and ultimately affect community assembly. Specifically, our goals were to: (1) the major plant functional traits at the community level to understand if they are significantly different with slope position, and if soil nutrients act as important dominant factors, and (2) to quantify the impact of slope position-mediated soil environmental filtering on the functional composition of shrub communities across three slope positions at hilly area. We anticipated that soil environmental factors vary along slope positions and can cause variability in plant functional traits in shrub communities. And environmental filtering became the major mechanism that maintained in this typical subtropical shrub community and would be helpful in the future to understand community assembly changes in Chinese hilly forest ecosystems.

## Materials and methods

### Study area

The study area was located on Jiangjiaba Hill, Guilin City, Southwest China (25°05′23″N, 110°17′32″E). The region belongs to the non-karst hill in Guilin, possesses laterite soil and ranges in altitudes from 100 to 500 m above sea level. Topography and altitude strongly influence microclimate and soil environmental factors, and therefore affect the distribution of plant species (Mantilla-Contreras et al., 2012). To measure such effects, we surveyed three slope positions (foot, middle, and upper) along an altitudinal transect. Based on our research, the upper slope had strong wind and shallow soils with little capacity for moisture retention that causes poor soil nutrients, while the slope foot had thick soil with a high soil water storage that was cooler, more humid, and had lower solar radiation. By comparison, the middle slope position had a relatively flat terrain with rich soil nutrients and moderate temperature, moisture, and light conditions (Liu et al., 2020). This region has a typical humid subtropical monsoon climate with noticeably dry and less rainy autumns and winters. Its annual temperature ranges from 17.8-19.1 °C with a frost-free period of 309 d and a mean annual sunshine duration of 1670 h (Data from the China Meteorogical Data Service Center; http://data.cma.cn). The coldest month (January) averages 8 °C and the hottest (August) averages 28 °C. The mean annual precipitation varies from 1814 to 1941 mm, with a distinct wet season from May to October (more than 80% of precipitation occurs during the wet season) and a dry season from November to April. The annual average evaporative capacity ranges from 1490 to 1905 mm, and the natural vegetation of this hilly region is dominated by shrub communities.

### Sampling design and field investigation

**Figure 1:**
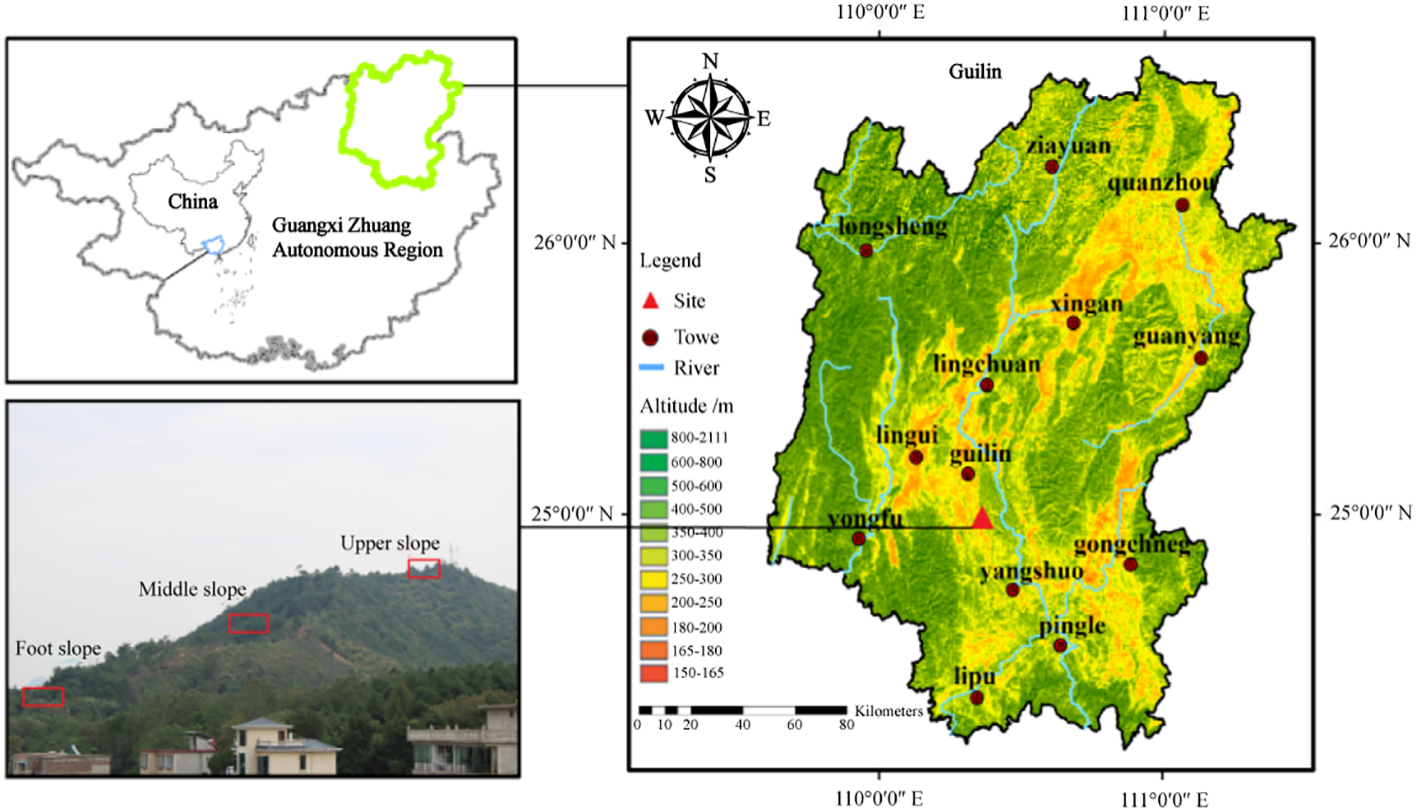
The geographical location of the study area and sampling sites in the hilly region, Guilin, Southwest China.

Data his study were collected in a typical hilly shrub community at the study site. Based on an initial field survey of woody shrubs, we established 108 10 m × 10 m plots with 36 plots each at the foot slope, middle slope, and upper slope from July to September 2016. The three positions are located at different points on a single slope (see **Figure 1** for details), and the plots in each slope are distributed continuously. In each sampling plot, all the woody individuals with basal diameter (BD) ≥ 1 cm were mapped, marked, and identified as species. We also reported topography (slope degree and position), dominant species, and abundance of evergreen and deciduous species at each slope position (**Table 1**). The nomenclature of each species were identified using the *Flora of China* (English edition; http://foc.iplant.cn).

**Table 1:** Topographic and ecological features of the three slope positions.

Slope Position	Number of Plots	Evergreen species	Deciduous species	Evergreen individuals	Deciduous individuals	Slope Degree Range (°)	Dominant Species
Foot	36	35	27	660	485	13–18	*Rhodomyrtus tomentosa* *Quercus fabri* *Ficus hirta* *Mallotus apelta*
Middle	36	26	23	463	561	7–12	*Quercus fabri* *Rhodomyrtus tomentosa* *Rhododendron molle* *Melastoma malabathricum*
Upper	36	21	17	387	410	17–21	*Schima superba* *Quercus fabri* *Melastoma malabathricum Rhodomyrtus tomentosa*

Our initial investigation documented 2966 live individuals of 75 tree species from 57 genera and 34 families. With each slope position, *Rhodomyrtus tomentosa* (Ait.) Hassk, *Quercus fabri* Hance, *Ficus hirta Vahl*, and *Mallotus apelta* (Lour.) Muell. Arg were the dominant species at the slope foot; *Quercus fabri* Hance*, Rhodomyrtus tomentosa* (Ait.) Hassk, *Rhododendron molle* (Blum) G. Don, and *Melastoma malabathricum* Linnaeus were dominant at the middle slope. The upper slope was mainly composed of *Schima superba* Gardn. et Champ, *Quercus fabri* Hance, *Melastoma malabathricum* Linnaeus, *and Rhodomyrtus tomentosa* (Ait.) Hassk. *Rhodomyrtus tomentosa*, *Ficus hirta*, *Mallotus apelta* and *Schima superba* are evergreen species, while *Quercus fabric*, *Rhododendron molle*, and *Melastoma malabathricum* are deciduous species that are among the most dominant species in each position.

### Measurement of functional traits

Four major functional traits were selected to characterize the ecological strategies of the plants at each different slope gradient: specific leaf area, leaf dry matter content, leaf chlorophyll content, and leaf thickness (Pérez-Harguindeguy et al., 2013; Jiang et al., 2015). For each individual, we collected three newly mature leaves from canopy branches that were completely exposed to sunlight, and avoided senescent or damaged leaves. The collected leaves were stored in sealed plastic bags with moistened paper towels and transported to the Key Laboratory of Ecology of Rare and Endangered Species and Environmental Protection at the Guangxi Normal University, Guilin, China, for further experiments. The leaf area was first measured by scanning fresh leaves using a Yaxin-1241 (Yaxin, Beijing, China). The mass of dried (72 h at 70 °C) leaf samples was then estimated with a precision of 0.1 mg and specific leaf area was calculated as leaf area divided by leaf dry mass. Leaf dry matter content was calculated from the oven-dry mass divided by fresh mass (Cornelissen et al., 2003; Pérez-Harguindeguy et al., 2013). Leaf thickness was measured by an electronic digital display caliper (SF2000, Guanglu, Guilin, China) with an accuracy of 0.01 mm and calculated as the mean thickness of three equally distanced points in the direction of the main veins. Leaf chlorophyll content was estimated using three values per lamina from a SPAD 502Plus meter (KONICA Minolta, Osaka, Japan).

### Measurement of soil environmental factors

Soil samples were collected between July and September 2016. According to the “S” type, we removed the leaf litter and humus layer, and five topsoil (0-15 cm depth) samples were collected from each 10 m × 10 m plot that were mixed to one 1000g sample for further analysis. We used the agricultural and chemical methods described by Bao (2000) to determine soil properties. Soil water content (SWC) was calculated as: SWC = (Massf - Massd)/Massd; where, Massf is the soil fresh mass and Massd is the soil dry mass. A digital pH meter (FE20K, Mettler-Toledo, Zurich, Switzerland) was used to measure soil pH using with a soil: to water ratio of 1:2.5. The soil organic content was measured using potassium dichromate oxidation method. Additionally, the total nitrogen was also determined by using automatic Kjeldahl analysis (KJELTEC™ 8400, FOSS Quality Assurance Co., Ltd., Hiller∅d, Denmark). The total phosphorus content of the soil was analyzed by acid digestion with an H_2_SO_4_ + HClO_4_ solution, and the total potassium was digested with an HF-HClO_4_-HNO_3_ acid mixture and determined by flame photometry. Additionally, the soil available nitrogen was quantified using the alkaline hydrolysis diffusion method, and the available phosphorus was measured using a molybdenum blue colorimeter after the sample had been extracted with 0.5 M Na_2_CO_3_. To measure soil available potassium, samples were shaken for 30 min with a 1 M ammonium acetate solution (1:10 *w/v*) and then analyzed by flame photometry. For each soil sample, the measurements were repeated three times and the average values were used for all subsequent soil physical and chemical analyses.

### Statistical analysis

The three slope positions (foot, middle, and upper) were used as groups for a One-way ANOVA comparison of the environmental factors, and Tukey’s HSD post-hoc comparisons were made to test for significant differences (family-wise error rate< 0.05) between groups. We coupled the individual traits to each individuals’ plot and then calculated CWM trait values. CWM values are proportional to species abundance and are more appropriate than unweighted means to characterize trait variation at the community level (Garnier et al., 2004; Lavorel et al., 2008). Here, we calculated CWMs with the “dbFD” function in the “*FD*” package of R software (Laliberté and Legendre, 2010; R Core Team, 2021), which is based on the Fdvar index:


$$CWM=\sum_{i=1}^{S}p_{i}\times trait_{i}$$

where *S* is the total number of species, *p_i_* is the relative abundance of the *i*th species and *trait_i_* is the trait value of the *i*th species.

To improve data normality and homoscedasticity, we rank-transformed the CWM values of the functional traits and environmental factors. We then used the One-way ANOVA and Tukey’s HSD post-hoc comparisons again to test how the CWM trait values varied among the three slope positions. We then performed redundancy analysis (RDA) to assess the correlations between plant functional traits and environmental variables across the three slope positions. From the trait and abundance data, we built a plot by trait matrix by averaging the trait values of each species weighted by their abundance in each plot. We then applied a forward selection that included the environmental variables and a Monte Carlo test with 999 unrestricted permutations to detect environmental factors that have a significant effect on trait variation.

To test the effects of modification of non-random assembly processes across the three slope positions, we adopted a trait-based null model approach. Here, the plant species found in each plot were treated as the local species pool, while all species observed across the 108 plots along the different slope positions were defined as the regional species pool. Following, the null distributions were built from a three-step procedure: 1) each species was randomly drawn without replacement from the species pool and repeated 999 times, 2) one species mean trait value derived from the regional functional species pool was randomly allocated to each previously selected species, and 3) the range of trait values in each plot (max-min) was calculated to generally predict the trait value distributions. By comparing the observed values of each plot with the expectation of the respective null model (Cornwell and Ackerly, 2009), we inferred random dispersion among local species trait values if the observed trait values were close to the expected distribution, while trait convergence was inferred if the range of the observed trait values was lower than expected, which would result from stronger environmental filtering. Trait divergence was also inferred if the range of the observed trait values was higher than expected, which would result from greater niche complementarity (greater niche complementarity; Lhotsky et al., 2016). We calculated the standardized effect size (SES; Gotelli and McCabe, 2002), implemented in the R package “*picante*” (Kembel et al., 2010) as:


$$SES=(Iobs-Inull)/SD_{null}$$

Where *I_obs_* is the observed value, *I_null_* is the mean of the null distributionand *SD_null_* is the standard deviation of null distribution in the plot. Positive and negative SES values indicate that the observed values are above and below the average expected value, respectively. The Wilcoxon signed rank test was then used to assess whether there were significant differences in random variation of functional trait values between plots at different slope positions.

## Results

### Changes in environmental factors across slope positions

**Figure 2:**
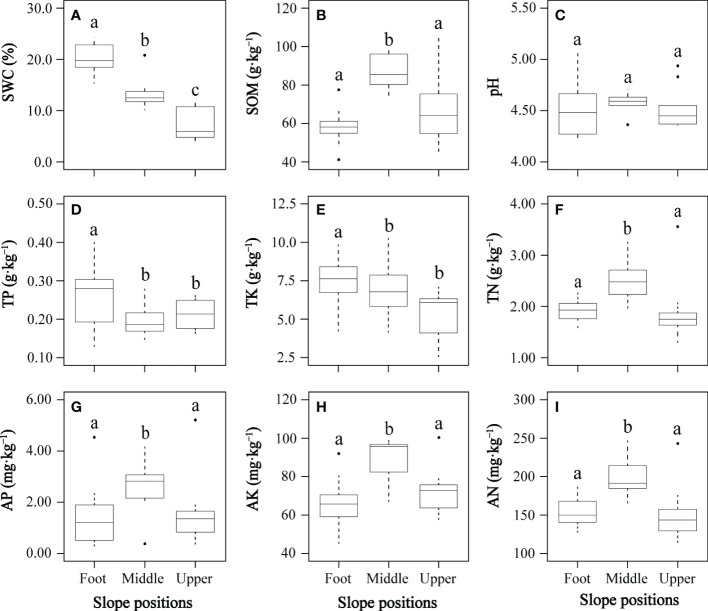
Changes of soil environmental factors **(A–I)** at three slope positions. AK, soil available potassium; AN, soil available nitrogen; AP, soil available phosphorus; pH, soil pH; SOM, soil organic content; SWC, soil water content; TK, soil total potassium; TN, soil total nitrogen; TP, soil total phosphorus. In each panel, boxes with different letters on top of the bars represent a significant difference at *P*< 0.05.

All soil environmental factors except soil pH differed significantly between at least two slope positions (**Figure 2**). Soil water content significantly decreased from slope foot to middle slope and from middle slope to upper slope (**Figure 2A**). The middle slope had significantly higher soil organic content, total nitrogen, available nitrogen, available phosphorus, and available potassium of than the slope foot and upper slope (**Figures 2B, F**–**I**, respectively). The slope foot had significantly higher total phosphorus and total potassium of soil than the middle and upper slopes (**Figures 2D, E**, respectively). The soil pH was slightly acidic to strongly acidic, and its soil organic matter, total nitrogen and available nitrogen were relatively rich, while soil total phosphorus, available phosphorus, total potassium and available potassium were relatively deficient, according to China’s second national soil census nutrient classification standard.

### Changes in community-level plant functional traits across slope positions

**Figure 3:**
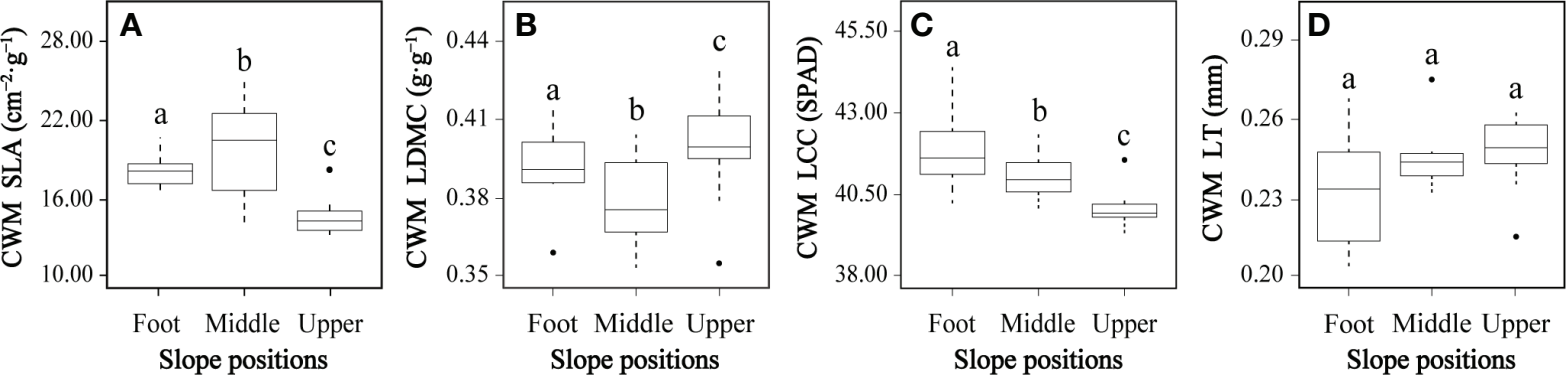
Community-weighted means (CMWs) of plant functional traits **(A–D)** at three slope positions. CWM SLA, CWM specific leaf area; CWM LDMC, CWM leaf dry matter content; CWM LCC, CWM leaf chlorophyll content; CWM LT, CWM leaf thickness. In each panel, boxes with different letters on top of the bars represent a significant difference at *P<* 0.05.

All CWMs of leaf functional traits except leaf thickness differed significantly between at least two slope positions (**Figure 3**). The CWM specific leaf area at the middle slope was significantly higher than both the slope foot and upper slope positions, and the slope foot CWM specific leaf area was significantly higher than the upper slope (**Figure 3A**). The CWM leaf dry matter content showed an inverted pattern; the middle slope was significantly lower than the slope foot, which was significantly lower than the upper slope (**Figure 3B**). The CWM leaf chlorophyll content significantly decreased from slope foot to middle slope and again from middle to upper slope (**Figure 3C**). There were no significant differences in CWM leaf thickness (**Figure 3D**).

### Relationships between community level plant functional traits and environmental factors across slope positions

**Figure 4:**
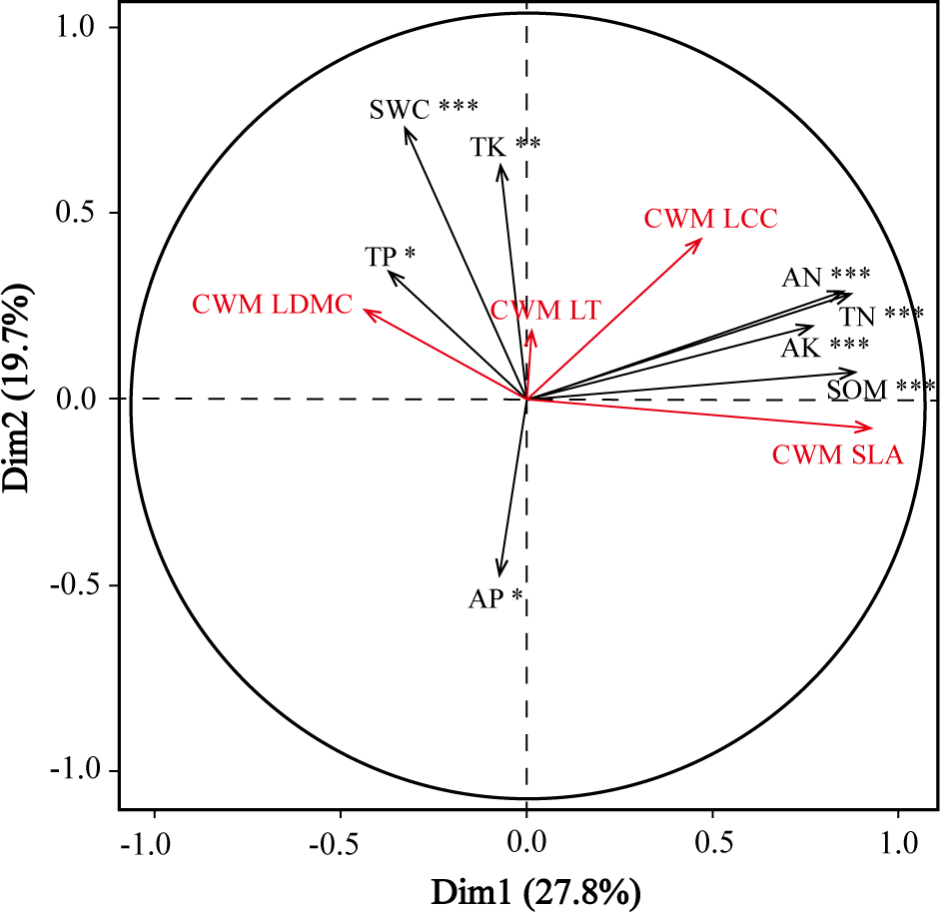
Redundancy analysis ordination diagram of the relationships between the four community-weighted means of functional traits (red) and eight selected soil environmental factors (black) across three slope positions. AK, soil available potassium; AN, soil available nitrogen; AP, soil available phosphorus; pH, soil pH; SOM, soil organic content; SWC, soil water content; TK, soil total potassium; TN, soil total nitrogen; TP, soil total phosphorus; CWM SLA, CWM specific leaf area; CWM LDMC, CWM leaf dry matter content; CWM LCC, CWM leaf chlorophyll content; CWM LT, CWM leaf thickness. *, **, and *** indicate significant correlation at *P*< 0.05, *P*< 0.01, and *P*< 0.001, respectively.

Redundancy analysis found significant relationships between community level plant functional traits and the environmental factors across the three slope positions (**Figure 4**). Soil environmental factors (available nitrogen, total nitrogen, available potassium, and organic content) significantly influenced the CWM specific leaf area and CWM leaf chlorophyll content, whereas soil total potassium, total phosphorus, and water content had significant effects on the CWM leaf dry matter content.

### Environmental filtering of traits across slope positions

**Figure 5:**
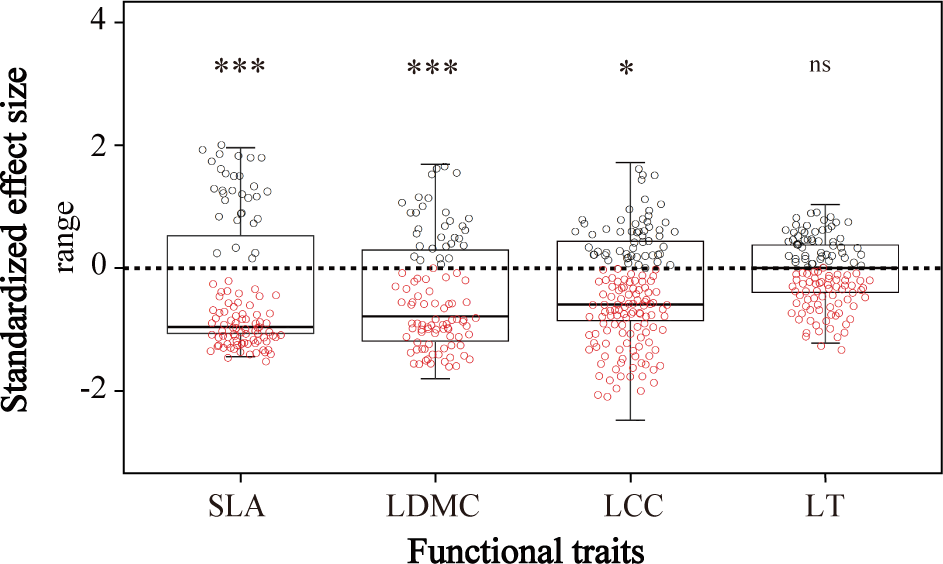
The standardized effect size of community-weighted means (CWMs) of plant functional traits between observed and randomly created communities. SLA, specific leaf area; LDMC, leaf dry matter content; LCC, leaf chlorophyll content; LT, leaf thickness. ns means no significant difference, * and *** indicate significant correlation at *P*< 0.05 and *P*< 0.001, respectively, by the Wilcoxon signed rank test.

By comparing to our null model expectations, we detected significant reductions in the community ranges of specific leaf area, leaf dry matter content, and leaf chlorophyll content (**Figure 5**)—indicating that environmental filtering had significant effects on plant functional trait values. We found no significant difference between the expected and observed distributions of leaf thickness, which suggested no environmental filtering of this trait (**Figure 5**).

## Discussion

### Relationships between community level plant functional traits and environment factors variation across slope positions

Slope position is an important topographic factor that encompasses substantial variation in micro-habitat conditions and which affects community-level plant functional traits (Méndez-Toribio et al., 2017). Consistent with a growing body of studies (e.g., Wang et al., 2016; Chelli et al., 2022), we found that SLA was negatively correlated with LDMC and observed the highest CWM specific leaf area with the lowest CWM leaf dry matter content. This fit our expectations for resource-rich environments, as the middle slope had high soil SOM, TN, AN, and AK, intermediate SWC, and a flatter terrain. In contrast, we found communities adapted to slope foot and upper slope conditions—generally resource poor environments—with low CWM specific leaf area and high CWM leaf dry matter content. Several studies have demonstrated that specific leaf area and leaf dry matter content can be useful proxies for resource capture and utilization (Fernanda et al., 2002; Shipley, 2006; Grotkopp and Rejmánek, 2007; Hodgson et al., 2011). The CWM leaf chlorophyll content significantly decreased from slope foot to middle slope to upper slope (**Figure 3C**), which may result, in part, from differences in species composition across slope positions. Most species found at the slope foot were evergreen and highly abundant (**Table 1**), indicating that evergreen species allocated greater resources to photosynthesis in this habitat with lower soil nutrients and weaker light (Liu et al., 2020). The middle slope position had intermediate CWM leaf chlorophyll content and more evergreen than deciduous species, but these evergreen species were less abundant. We had predicted the CWM leaf chlorophyll content of the middle slope to be highest because of its high nutrient richness. The unexpected result that it was intermediate may be due to two reasons: first, a decrease in soil water content could inhibit chlorophyll biosynthesis leading to relatively low of leaf chlorophyll content (He et al., 2022); second, high available potassium, total nitrogen, and available nitrogen of soil may increase net photosynthetic rate (Hu et al., 2017).

### Environmental filtering for plants with different functional traits

The observed ranges of specific leaf area, leaf dry matter content, and leaf chlorophyll content (but not leaf thickness) were significantly smaller than expected based on our null models, suggesting that environmental factors significantly filter the CWM specific leaf area, leaf dry matter content, and leaf chlorophyll content (**Figure 5**). We showed that soil nutrients and water availability strongly restricted these CWM trait values across the three slope positions, as evidenced by the collinearity of these features with CWMs in the RDA ordination (**Figure 4**). Local topography (i.e., slope position) can therefore drive functional trait values, mostly through indirect effects of soil nutrients and soil water content. At our study site, the upper slopes were steep and the soils infertile; the upper slope had less developed soil, less soil water, and fewer soil nutrients, while the slope foot had relatively thick soil with higher soil water storage, lower solar radiation, and was cooler and more humid. The middle slope position was relatively flat with fertile soil and mild temperature, moisture, and light conditions. The species composition changed from mostly evergreen, shade-intolerant species with relatively low nutrient demand at the slope foot and upper slope positions to deciduous species with high nutrient demands at the middle slope position. Fertile soils and mild climatic conditions had strong filtering effects; deciduous shrub species with high specific leaf area, low leaf dry matter content, and moderate leaf chlorophyll content dominated at the middle slope position, whereas evergreen species with low specific leaf area and high leaf dry matter content dominated in slope positions with infertile soils, steeper slopes, and more extreme soil water contents. Our null model approach allowed us to detect patterns of habitat filtering, which differed between traits.

## Conclusion

Our results showed that multiple environmental factors and the CWMs of specific leaf area, leaf dry matter content, and leaf chlorophyll content were significantly different between slope positions and that most environmental factors examined had significant effects on plant functional traits. Together, these findings indicate that slope position gradients—with varying soil nutrient and climatic conditions—have significant effects on plant functional trait variation. In summary, the detection of habitat filtering depends critically on the type of trait considered: different traits, which reflects different aspects of plant physiology, may be differentially filtered by the environment.

## Data availability statement

The original contributions presented in the study are included in the article/supplementary materials, further inquiries can be directed to the corresponding author/s.

## Author contributions

YJ designed and oversaw the study. KC, YP, Y.L, YH, WZ, HL, JC, and YF collected the field and laboratory data. KC, YP and YJ conducted the statistical analyses and wrote the first draft of the manuscript. All authors contributed to the article and approved the submitted version.
